# Menopause Characteristics, Total Reproductive Years, and Risk of Cardiovascular Disease Among Chinese Women

**DOI:** 10.1161/CIRCOUTCOMES.117.004235

**Published:** 2017-11-08

**Authors:** Ling Yang, Liling Lin, Christiana Kartsonaki, Yu Guo, Yiping Chen, Zheng Bian, Kaixu Xie, Donghui Jin, Liming Li, Jun Lv, Zhengming Chen

**Affiliations:** From the Medical Research Council Population Health Research Unit (L.Y., C.K., Y.C.) and Clinical Trial Service Unit and Epidemiological Studies Unit (L.Y., C.K., Y.C., Z.C.), University of Oxford, United Kingdom; Department of Epidemiology and Biostatistics, School of Public Health, Peking University Health Science Center, Beijing, China (L. Lin, L. Li, J.L.); Chinese Academy of Medical Sciences, Beijing (Y.G., Z.B.); NCDs Prevention and Control Department, Tongxiang CDC, China (K.X.); and NCDs Prevention and Control Department, Hunan CDC, Changsha, China (D.J.).

**Keywords:** cardiovascular disease, China, female, postmenopause, stroke

## Abstract

**Background—:**

Previous studies, mostly of Western women, have reported inconsistent findings on the association of menopause characteristics (status, age, and time since menopause) and total reproductive years with risk of cardiovascular disease (CVD).

**Methods and Results—:**

The China Kadoorie Biobank recruited 302 632 women in 2004 to 2008 from 10 regions across China. During 9-year follow-up, 19 393 incident cases of stroke, 18 611 of ischemic heart disease, and 4978 CVD deaths occurred. Cox regression yielded adjusted hazard ratios relating each menopause characteristic and total reproductive years to CVD risk. Among 274 233 women with no prior CVD at baseline, 134 010 were naturally postmenopausal women (mean [SD] age at menopause of 48.6 [4.0] years and total reproductive years 32.7 [4.4]). Compared with premenopausal women, naturally peri- or postmenopausal women were at a higher risk of either fatal or nonfatal CVD. Among women who had had menopause, inverse associations were observed between age at menopause and risks of CVD mortality, incident ischemic heart disease, stroke, and subtypes of stroke, with 1.5% higher risk of CVD death (*P*<0.001), 0.7% for incident ischemic heart disease (*P*=0.002), and 0.5% for incident stroke (*P*=0.02) for every 1 year lower age at menopause. Compared with women who had menopause at age 48 to 50 years, lower age at menopause (ie, <43 years) was associated with 14% higher risk of CVD death and 6% higher risks of both incident ischemic heart disease and stroke. Higher risks of both fatal and nonfatal CVD were also found in women with 5 to 10, 10 to 15, 15 to 20, or >20 years since menopause compared with <5 years since menopause. Total reproductive years were inversely associated with risks of both fatal and nonfatal CVD, with 1.4% lower risk of CVD death per additional reproductive year (*P*<0.001).

**Conclusions—:**

Women with younger age at menopause, longer time since menopause, or fewer total reproductive years had a higher risk of CVD.

## Abstract 2

Supplemental Digital Content is available in the text.

**WHAT IS KNOWN**

- Previous studies, mostly of Western women, have reported inconsistent findings on the association of age at menopause with risk of cardiovascular disease (CVD).
- Only a few studies have examined the association between the time since menopause or total reproductive years and risk of CVD.

**WHAT THE STUDY ADDS**

- This large study of Chinese women found that age at menopause and total reproductive years were inversely associated with risks of both fatal and nonfatal CVD, as well as with both ischemic and hemorrhagic stroke subtypes.
- There was a higher risk of CVD mortality among Chinese women with early menopause.
- The risks for CVD were higher with longer time since menopause among postmenopausal women.

Cardiovascular disease (CVD) is a dominant cause of mortality and morbidity throughout the world and the leading cause of death and disability in China.^1^ However, both incidence and mortality of CVD are low in women of reproductive age but rise to a significant level among postmenopausal women, for example, the risk of stroke was reported to be approximately double in women during the 10 years after menopause.^2^ The menopausal transition is associated with significant hormonal changes; most importantly, oestradiol levels decline by ≈60%, and the levels continue to decrease and then plateau 1 to 3 years post-menopause with, overall, a 7- to 10-fold reduction in oestradiol levels from pre- to post-menopause.^2^ Early decline in oestradiol levels related to early age at menopause could be detrimental to the health of bones and blood vessels and may thus be associated with risk of CVD.^3–8^ However, age at menopause is not the only determinant of endogenous oestrogen exposure. Other reproductive factors, such as age at menarche, pregnancy consequences, and duration of breastfeeding, may also determine the duration and level of exposure to endogenous estrogen.^9–11^ Given the effects of oestrogen on lipoproteins, blood pressure, and other cardiovascular risk factors, these factors and the duration of time since menopause may also influence CVD risks in postmenopausal women.^9^ It is, however, not clear to what extent postmenopausal women may benefit from the long-term effects of their premenopausal endogenous oestrogen exposure. Given the logistical difficulty in measuring endogenous hormones during the life course, epidemiological studies focused on the association between proxies for exposure to endogenous hormones, such as age at menopause, time since menopause, and total reproductive years (ie, the duration between menarche and menopause), with CVD risk. The majority of previous studies have been conducted in Western populations, with inconsistent findings reported. It is of particular interest to investigate the associations among Chinese women, a population with unique reproductive histories because of the country’s One Child Policy, which was introduced in 1979 and which may have long-term consequences on major health outcomes and their risk factors.^12,13^ In the present study, we aim to investigate the association between various menopause characteristics (status, age, and time since menopause), total reproductive years, and the risk of both fatal and nonfatal CVD in a single large cohort study (China Kadoorie Biobank) involving 303 000 Chinese women.

## Methods

### Baseline Survey

Details of the China Kadoorie Biobank study population have been reported previously.^14^ Briefly, the baseline survey was conducted in 2004 to 2008 in 10 geographically defined areas in China. Data about general demographic and socioeconomic status and dietary and other lifestyle habits (eg, smoking, alcohol drinking, and physical activity) were collected using an interviewer-administered laptop-based questionnaire. The medical history included questions on whether participants had ever been diagnosed with a range of chronic diseases (eg, heart disease, stroke, diabetes mellitus, and cancer). Women were also asked about their reproductive history, including age at menarche, parity, age at birth and breastfeeding duration for each live birth, menopausal status (age at menopause for those postmenopausal women), and the history of use of oral contraceptives (OC). Physical measurements included blood pressure and anthropometry. All participants provided a blood sample and provided written consent for follow-up. International, national, and local ethics approval was obtained.

### Follow-Up for Mortality and Morbidity

Participants were followed-up for cause-specific morbidity and mortality through linkage with regional disease and death registers and for all hospitalized events through electronic data linkage with the nationwide health insurance system. To minimize losses to follow-up, active follow-up (ie, visiting the local community or directly contacting participants) was also performed annually.^14^ Fatal and nonfatal events were coded according to *International Classification of Diseases, Tenth Revision*, blinded to baseline information. The primary outcomes examined for the present study were CVD death (I00-25, I27-88, I95-99) and the incidence of ischemic heart disease (IHD: I20-25) and stroke (I60-I61, I63-I64) or subtypes of stroke (hemorrhagic [I61] and ischemic [I63] stroke).

### Statistical Methods

From a total of 302 632 women recruited at baseline, we excluded participants who had missing, inconsistent, or implausible values for reproductive factors (n=930), reported a prior history of CVD or cancer, or had hysterectomies or one or both ovaries removed (n=27 469), leaving 274 233 women in the main analyses.

Cox proportional hazards models were used to estimate hazard ratios (HRs) for the associations between menopause characteristics (status [ie, pre-, peri- or postmenopausal, ie, those who reported at baseline that they had not, were currently under, or already had natural menopause], age at menopause, time since menopause [defined as the years between age at menopause and age at risk of CVD event]), total reproductive years, and risks of fatal or nonfatal CVD. The analysis for menopausal status was conducted among all women, and other analyses were confined to postmenopausal women only. Analyses were conducted using time since entry into the study as the time scale and were stratified by (time varying) age at risk (except for time since menopause) and region to allow each age group and region to have a different baseline hazard rate for the disease. Based on prior knowledge, the following time-invariant covariates were adjusted for in the model: highest level of education, household income, smoking, alcohol drinking, measured blood pressure, body mass index, physical activity (Metabolic Equivalents of Task h/d),^15^ prevalent diabetes mellitus status, and other reproductive factors (age at menarche [except in analyses for total reproductive years]), parity, number of abortions, age at first birth, breastfeeding duration per child, OC use status). For the analyses for time since menopause, age was used as the underlying time scale with time since menopause (in 5-year groups) as a time-varying variable. Associations were compared between subgroups of women defined by region, education, birth cohort, smoking status, alcohol drinking status, hypertension history, body mass index, and other reproductive factors (eg, age at menarche, number of live birth, abortion status, and, for parous women, age at first birth and duration of breastfeeding per child). Sensitivity analyses were conducted (1) among those who never smoked, drank alcohol, or used OC; and (2) among women aged ≥57 years (of whom 99% reported being post-menopausal) to avoid any potential distortion of the distribution of age of menopause among younger women. The 95% confidence intervals for each log HR were estimated using the floating absolute risk method, which facilitates comparisons between different categories of the exposure, rather than only between one arbitrarily chosen reference group and each of the other categories.^16^ To adjust for regression dilution bias related to reporting error in age at menopause or reproductive years, HRs in the groups determined at baseline were plotted against the mean value of age at menopause or reproductive years in that group at the 2008 resurvey.^17,18^ Analyses were performed using SAS version 9.3 and R version 3.3.1.

## Results

Among the 274 233 women included, the mean (SD) age was 50.4 (10.4) years, 42.9% were urban residents, and few ever smoked (2.3%), drank alcohol regularly (2.1%), or used OC (9.7%). Nearly all women had given birth (98.7%) and most parous women breastfed their children (97.3%). At baseline, 45.9% of the women were premenopausal, 5.2% peri-menopausal, and 48.9% postmenopausal. Compared with premenopausal women, naturally peri- or postmenopausal women tended to be less educated and less physically active, with higher mean blood pressure and higher prevalence of smoking, alcohol drinking, overweight/obesity, and to have a greater number of children, higher age at menarche, and longer breastfeeding duration (Table 1). Among 134 010 naturally postmenopausal women at baseline, the overall mean age at menopause was 48.6 (4.0) years, and the mean total reproductive years was 32.7 (4.4) years. Compared with women with a higher age at menopause or more total reproductive years, respectively, women with earlier menopause or fewer reproductive years were, on average, slightly younger and leaner at baseline, more likely to be resident in rural areas, less educated, smoke more, and be more active, and had, on average, lower blood pressure, with a higher proportion of nulliparity and lack of breastfeeding and a lower mean of breastfeeding duration (Table 2).

**Table 1.**
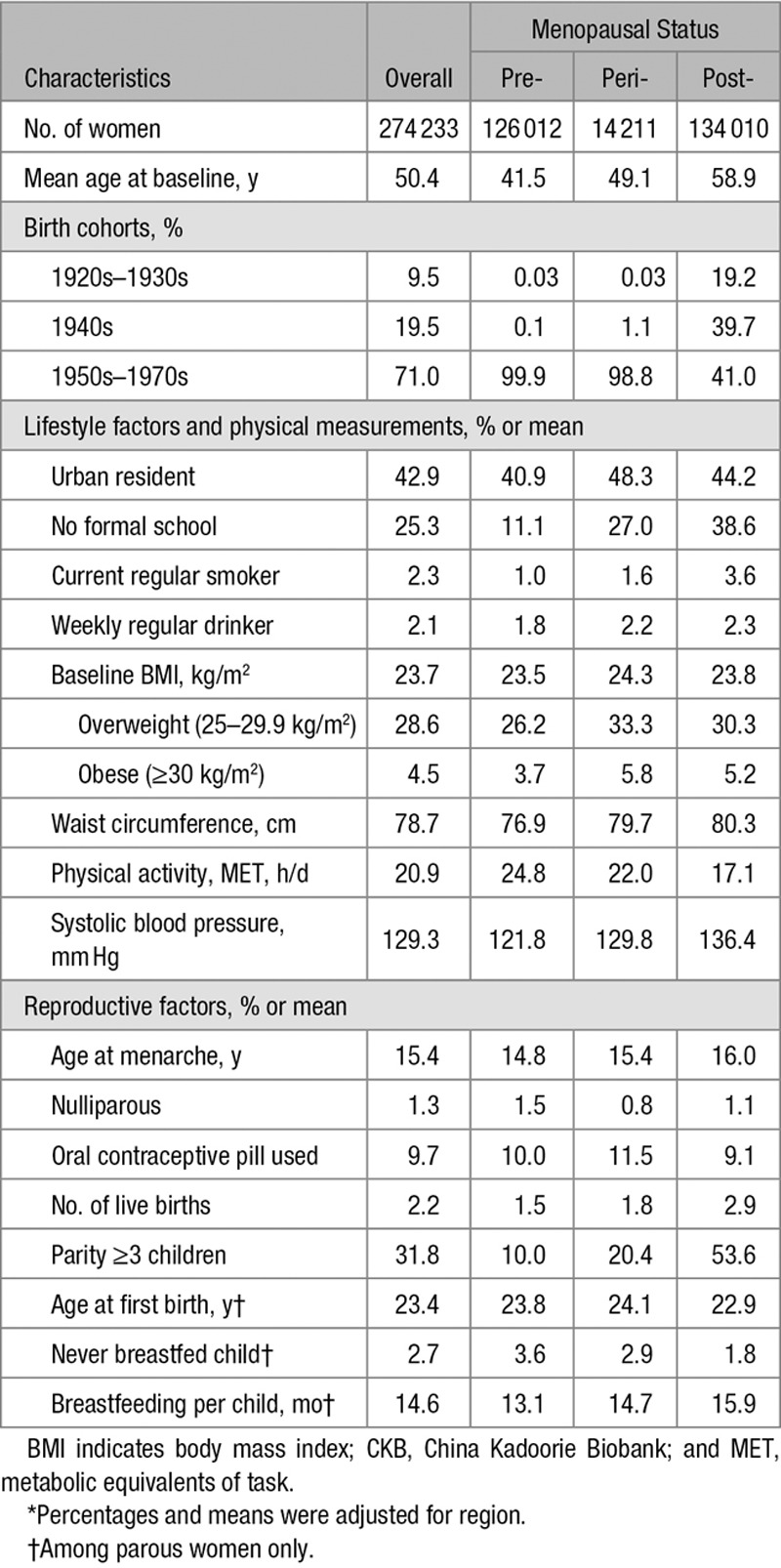
Baseline Characteristics of the CKB Women*

**Table 2.**
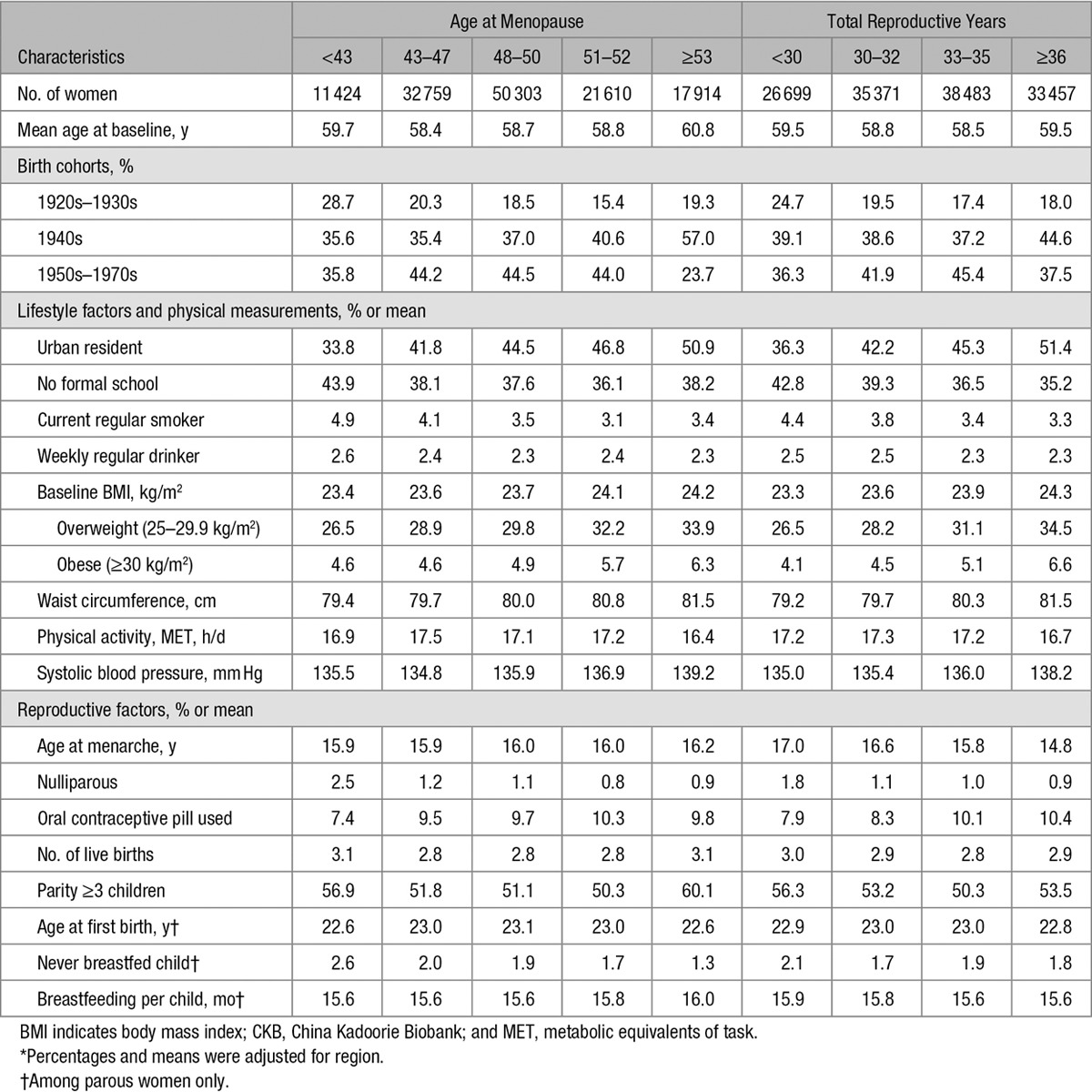
Basic Characteristics for the CKB Nature Postmenopausal Women*

By the end of 2015 (≈2.5 million person-years of follow-up), overall 19 393 participants had a stroke (including 15 454 ischemic, 2761 hemorrhagic), 18 611 had IHD, and 4978 died from CVD (among postmenopausal women, 15 252 had a stroke [12 253 ischemic, 2125 hemorrhagic] and 14 248 IHD, and there were 4524 CVD deaths).

### Menopausal Status

**Figure 1.**
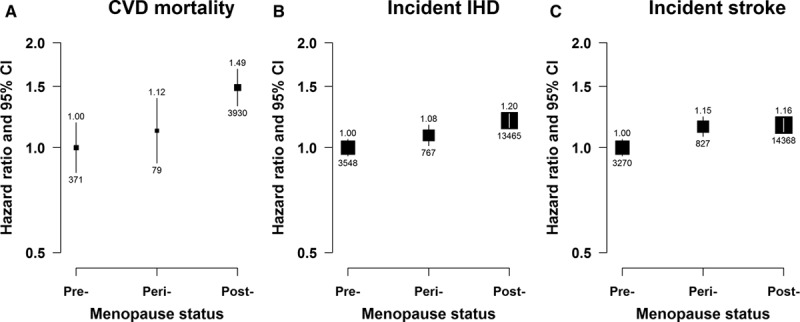
Adjusted hazard ratios (HRs) for total cardiovascular disease (CVD) mortality, incident ischemic heart disease (IHD), and incident stroke by menopause status. **A**, CVD mortality. **B**, Incident IHD. **C**, Incident stroke. Models were stratified by age and study area and adjusted for education, household income, smoking, alcohol drinking, blood pressure, body mass index, physical activity, prior diabetes mellitus status, age at menarche, parity, abortion, age at first birth, breastfeeding duration per child, and oral contraceptive use. Premenopausal women were used as the reference category. Squares represent the HR with area inversely proportional to the variance of the log HR. Vertical lines indicate the corresponding 95% confidence intervals (CIs). The numbers above the CIs are HRs and those below are the numbers of events studied.

After adjustment for potential confounding lifestyle risk factors and other reproductive factors, compared with premenopausal women, postmenopausal women had statistically significantly higher risks of both fatal and nonfatal CVD with HR of 1.49 (95% confidence interval: 1.32–1.68) for CVD mortality, 1.20 (1.14–1.25) for incident IHD, and 1.16 (1.11–1.21) for incident stroke (Figure 1). Increased risks, although of a slightly lower magnitude, were also found in perimenopausal women.

### Age at Menopause

**Figure 2.**
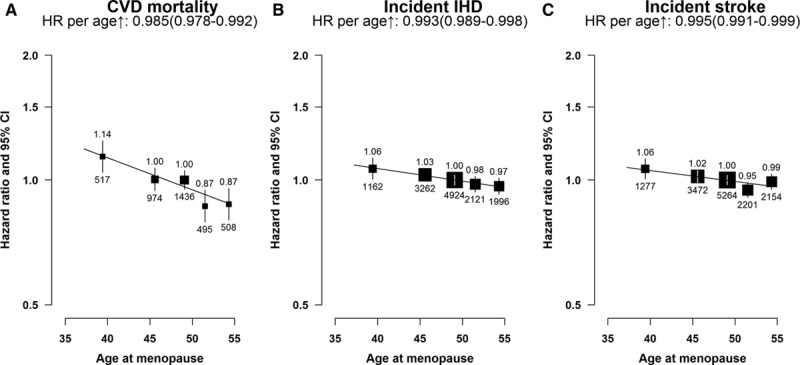
Adjusted hazard ratios (HRs) for total cardiovascular disease (CVD) mortality, incident ischemic heart disease (IHD), and incident stroke vs age at menopause (years), among postmenopausal women only. **A**, CVD mortality. **B**, Incident IHD. **C**, Incident stroke. Women who had menopause at age 48 to 50 y were used as the reference category. The HRs are plotted against mean age at menarche at resurvey for each category. Squares represent the HR with area inversely proportional to the variance of the log HR. Vertical lines indicate the corresponding 95% confidence intervals (CIs). The numbers above the CIs are HRs and those below are the numbers of events studied.

**Figure 3.**
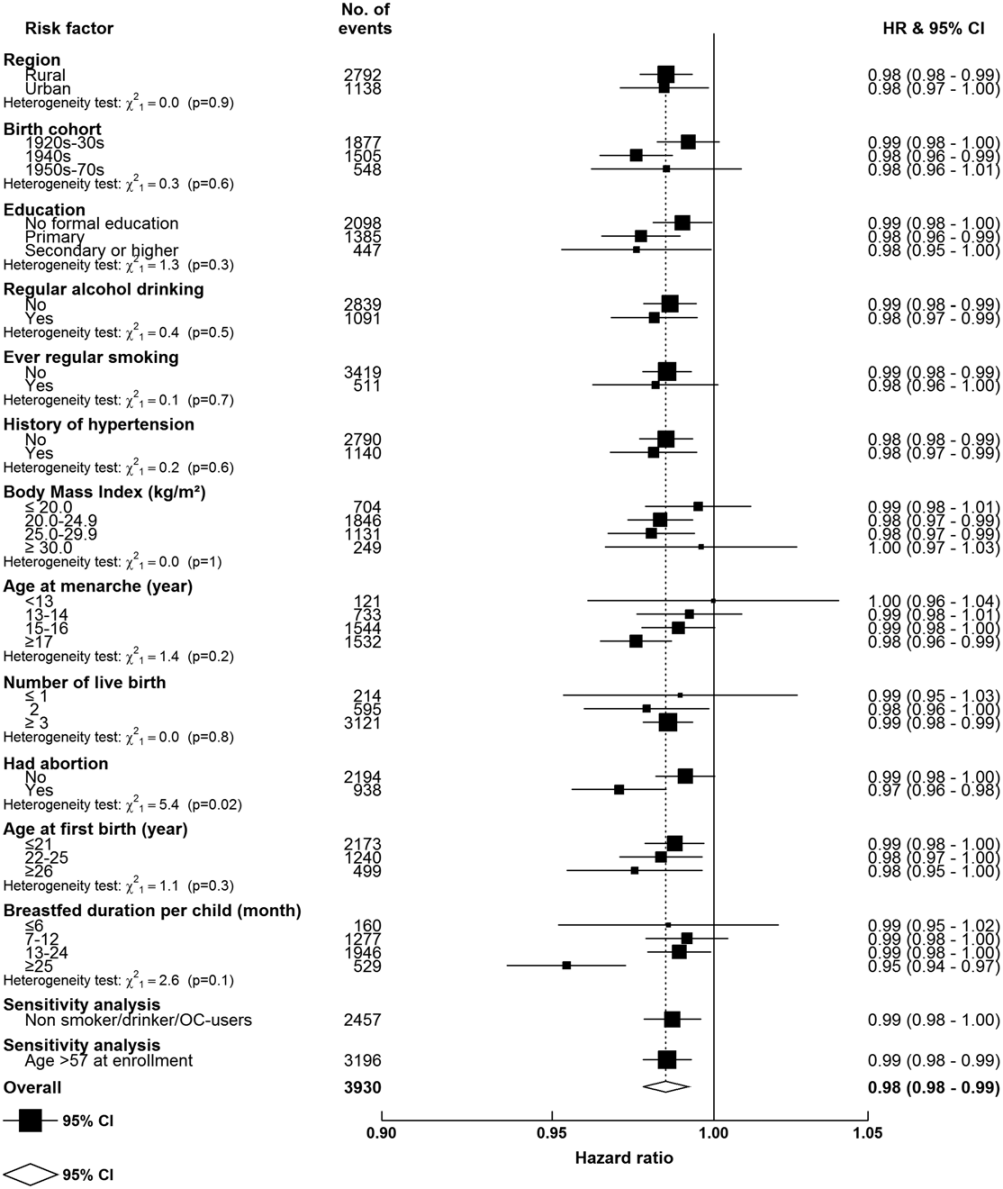
Adjusted hazard ratios (HRs) for cardiovascular disease mortality per year increase in age at menopause, within various subgroups, among postmenopausal women only. Models were stratified by age and study area and adjusted for education, household income, smoking, alcohol drinking, blood pressure, body mass index, physical activity, prior diabetes mellitus status, age at menarche, parity, abortion, age at first birth, breastfeeding duration per child, and oral contraceptive use. The dotted vertical line indicates the overall change of every 1 y greater age at menarche; the open diamond indicates this estimate and its 95% confidence interval (CI). Sensitivity analyses were among (1) women who never smoked, drank alcohol, or used oral contraceptives and (2) women who were aged >57 y at enrollment.

Overall, among postmenopausal women, a statistically significant inverse association was observed between age at menopause and risk of CVD mortality (Figure 2A), incident IHD (Figure 2B) and incident stroke (Figure 2C), and subtype of stroke (Figure I in the Data Supplement). For each year lower age at menopause, there was a 1.5% higher risk of CVD mortality (*P*<0.001), 0.7% higher risk of incident IHD (*P*=0.002), and 0.5% higher risk of incident stroke (*P*=0.02). Compared with women who had menopause at age 48 to 50 years, women with a lower age at menopause (ie, <43 years) had a 14% higher risk of CVD death and ≈6% higher risks of incident IHD and stroke (Figure 2). Although the magnitude of the associations varied slightly, the above described associations between age at menopause and fatal or nonfatal CVD risk were broadly consistent across different population subgroups defined by region and by sociodemographic, lifestyle, or other reproductive factors or in sensitivity analyses among the subset of women who never smoked, drank alcohol, or used OCs. However, the inverse linear relationship with CVD deaths was more pronounced among women who breastfed for >2 years per child than in parous women who breastfed for a shorter time period (*P*_for heterogeneity_=0.005) and in women who had an abortion compared with those who had not (*P*_for heterogeneity_=0.02; Figure 3).

### Time Since Menopause

**Figure 4.**
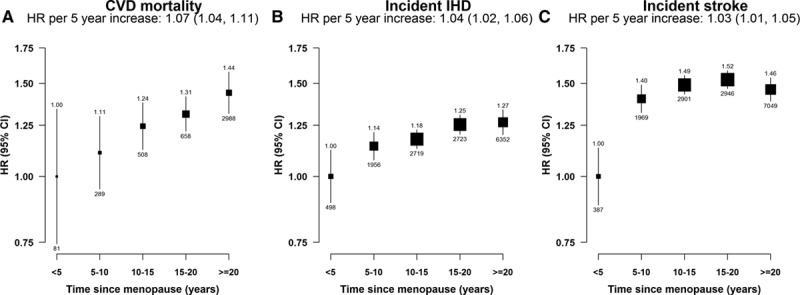
Adjusted hazard ratios (HRs) for total cardiovascular disease (CVD) mortality, incident ischemic heart disease (IHD), and incident stroke by time since menopause, among postmenopausal women only. **A**, CVD mortality. **B**, Incident IHD. **C**, Incident stroke. Models were stratified by age and study area and adjusted for education, household income, smoking, alcohol drinking, blood pressure, body mass index, physical activity, prior diabetes mellitus status, age at menarche, parity, abortion, age at first birth, breastfeeding duration per child, and oral contraceptive use. Premenopausal women were used as the reference category. Squares represent the HR with area inversely proportional to the variance of the log HR. Vertical lines indicate the corresponding 95% confidence intervals (CIs). The numbers above the CIs are HRs and those below are the numbers of events studied.

Compared with women who were <5 years postmenopausal, higher risks of CVD mortality, incident IHD, and stroke were observed among women with 5 to 10, 11 to 15, 16 to 20, or >20 years post-menopause. There was a 7% (4%–11%) higher risk of CVD death and a 4% (2%–6%) higher risk of incident IHD per 5 additional years since menopause (*P*<0.001). A significantly higher risk of stroke (HR=1.40; 1.32–1.49) was observed in women 5 to 10 years after menopause compared with those with <5 years since menopause. However, the risk leveled off at time since menopause >10 years (Figure 4).

### Total Reproductive Years

**Figure 5.**
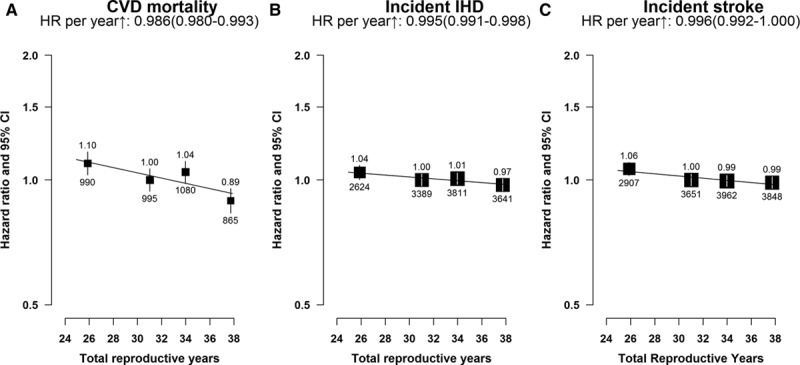
Adjusted hazard ratios (HRs) for total cardiovascular disease (CVD) mortality, incident ischemic heart disease (IHD), and incident stroke by total reproductive years. **A**, CVD mortality. **B**, Incident IHD. **C**, Incident stroke. Models were stratified by age and study area and adjusted for education, household income, smoking, alcohol drinking, blood pressure, body mass index, physical activity, prior diabetes mellitus status, parity, abortion, age at first birth, breastfeeding duration per child, and oral contraceptive use status. Women with 30 to 32 total reproductive years were used as the reference category. Squares represent the HR with area inversely proportional to the variance of the log HR. Vertical lines indicate the corresponding 95% confidence intervals (CIs). The numbers above the CIs are HRs and those below are the numbers of events studied.

The number of total reproductive years was inversely associated with the risk of CVD and statistically significantly associated with lower fatal CVD risk (1.4% lower risk of CVD death per longer reproductive year [*P*<0.001]) and nonfatal IHD, stroke, or stroke subtypes (Figure 5; Figure II in the Data Supplement). Compared with women with 30 to 32 total reproductive years, those with a shorter reproductive span tended to have a slightly higher risk of CVD mortality (10%) and incident stroke and IHD (≈5%). A lower risk for CVD mortality was found among women who had an abortion or who breastfed for >2 years per child. Nevertheless, the inverse association did not differ substantially when analyses were undertaken among subgroups of women defined by various sociodemographic, lifestyle, or other reproductive factors, nor in the subset of women who never smoked, drank alcohol, or used OCs, or in elderly women (Figure III in the Data Supplement).

## Discussion

Based on 274 233 middle-aged women recruited from 10 regions of China who had no prior history of CVD, postmenopausal women were at a higher risk of both fatal and nonfatal CVD compared with premenopausal women of the same age. Inverse trends in risk for both age at menopause and total reproductive years were consistently observed among naturally postmenopausal women although the magnitude of the associations was much stronger for fatal than for nonfatal CVD. The inverse associations were broadly consistent across most subgroups of women. Longer time since menopause was associated with higher risks of both fatal and nonfatal CVD. To our knowledge, this is the first large prospective study that has comprehensively investigated menopause characteristics with the risk of CVD.

The present study confirmed the higher risk of CVD observed among postmenopausal women compared with premenopausal women.^3^ Previous studies, however, reported inconsistent findings on the association between age at menopause and risk of CVD. This discrepancy may be partly because of small sample sizes, residual confounding from unmeasured or other potential risk factors (eg, other reproductive factors), or the different age ranges of study populations. The higher risk of CVD mortality among women with early menopause in the present study was generally in line with recently published studies conducted in Western women.^19–22^ Based on 5 studies of 65 653 women with 6979 CVD deaths, a meta-analysis reported a 19% (8%–31%) higher risk of overall CVD mortality among women who experienced menopause before 45 years versus those who experienced it at older ages.^20^ However, the inverse association appeared to be more pronounced in younger than in older generations; this effect was observed in our study but was not statistically significant.^7,23,24^ A null association between age at menopause and CVD mortality was reported in Asian studies, including 2 Chinese prospective studies conducted in Shanghai.^5,8,23,25^ The reasons for the more pronounced inverse association among women who had had abortion or who breastfed >2 years per child were not clear, and further investigation is needed. Few previous studies have investigated incident CVD, and instead, an increased risk of IHD among women with early menopause has often been presented.^26–28^ A meta-analysis, including 5 studies among ≈50 000 Western women, showed a high risk of incident CVD comparing age at menopause <45 versus ≥45 years (pooled HR 1.50 [1.28–1.76] for IHD, 1.23 [0.98–1.53] for stroke) but no statistically significant association when comparing age at menopause >50 versus 45 to 49 years (1.12 [0.95–1.31] for IHD, 0.95 [0.74–1.23] for stroke).^20^ In terms of subtypes of stroke, the association tended to be restricted to ischemic stroke.^29^ A study conducted in 4790 Japanese women showed that the association of early menopause was stronger for ischemic stroke than for all stroke subtypes combined (157% versus 56% increasing).^30^ In the Framingham study, among 1430 women with 234 ischemic strokes identified over 22 years of follow-up, women with menopause before age 42 years had twice the risk of ischemic stroke (HR=2.03 [1.16–3.56]) compared with all other women.^6^ In the present study, a weak inverse association was consistently shown between age at menopause and both ischemic and hemorrhagic strokes, and, more interestingly, the magnitude of association was somewhat stronger for hemorrhagic stroke. The pathological mechanisms of ischemic and hemorrhagic strokes are different, thus further research to explore the effects of age at menopause on different stroke subtypes is required.

The inconsistent findings on the associations between age at menopause and CVD risk among different age groups may also be because of the longer period of time since menopause among older women. A few studies have suggested that time since menopause may associate with some conventional CVD risk factors, such as metabolic syndrome or obesity.^31–34^ However, only 2 studies investigated the association between time since menopause and CVD risk, in which opposite results were presented. No increased risk of myocardial infarction in postmenopausal women <10, 10 to 20, or >20 years post-menopause, compared with pre- or perimenopausal women, was found in a case–control study (429 cases and 863 controls) in Italy.^32^ In a Chinese population study, women were at a greater risk of CVD 2 to 6 years after menopause than at <1 year post-menopause, with evidence of an increase in risk after 6 years.^33^ Nevertheless, because the majority (70%) of women were <6 years post-menopause, the long-term effect from the time since menopause could not be shown. The consistently increased risk of both fatal and nonfatal CVD found in the present prospective study provides, for the first time, evidence on the association of time since menopause with CVD risk. Further investigation of the associations between time since menopause and other intermediate risk factors or relevant diseases is needed to elucidate the biological mechanisms.

Only a few studies have examined the association between total reproductive years and CVD risk. These have mostly investigated fatal outcomes and have reported no significant results, which is inconsistent with our findings.^8,23,25^ The differences in the magnitude of association of age at menopause and total reproductive years with nonfatal and fatal CVD may be because of the severity and misclassification of end points studied. Fatal CVD end points are generally more accurate and may include more severe CVD cases than nonfatal outcomes. Using only fatal CVD cases may reflect the association of reproductive factors with severe CVD phenotypes. However, nonfatal end points may be contaminated with some mild disease events that may attenuate the association of interest. Further event adjudication of nonfatal events may help to clarify the association.

Several plausible mechanisms were postulated to explain the effect of endogenous estrogen on CVD pathogenesis, including direct effects on the vascular wall and long-term indirect effects via improvements in CVD risk factors.^2–4^ Loss of ovarian function results in a low level of 17b-oestradiol, which reduces the development of early atherosclerotic lesions. Nevertheless, it is possible that postmenopausal women still benefit from the endogenous 17b-oestradiol produced during their reproductive years because of the effects exerted on conventional CVD risk factors.^35^ With comprehensive adjustment for women’s other reproductive factors and lifestyle risk factors, results from the present study generally support the above hypothesis. Studies have shown that heredity seems to be the most important determinant of age at menopause.^36^ A study of sister pairs showed that ≈85% of the phenotypic variation in age at menopause was genetically determined.^37^ However, limited evidence comes from Chinese studies.

Important strengths of our study are the wide variation in values of the major lifestyle and reproductive factors of interest and the large number of person-years in each category, providing sufficient power to detect associations. The study has several other strengths, including standardized approaches and stringent quality control for data collection and good reproducibility on a comprehensive range of information, including both lifestyle and reproductive factors.^12,13^ These allow us to simultaneously adjust for potential confounders, including other reproductive factors during women’s reproductive years, to reliably assess the associations. Several limitations need to be taken into account. Using exogenous female hormones may affect the association between endogenous hormones and disease risk, however, unlike women in the West, only a minority of Chinese women used exogenous hormones.^38^ Misclassification of diseases, particularly for nonfatal CVD, would have attenuated the results toward the null. The potential recall bias about self-reported reproductive factors, particularly age at menarche that was recalled decades later, is likely to be small because of the high repeatability obtained, for example, the intraclass correlation coefficient of 0.84 for age at menarche recorded between the baseline survey and the resurvey.^12^ Furthermore, although we have allowed for a comprehensive range of potential confounders, residual confounding from other known or unknown risk factors may still exist because of the observational nature of the study.

This large study of Chinese women found that age at menopause and total reproductive years were inversely associated with both fatal and nonfatal CVD while the disease risks were higher with longer time since menopause. Further studies are needed to confirm these findings and to clarify the mechanisms that link these reproductive exposures to CVD risk. With a better understanding of the impact of reproductive factors on disease risk, our findings may be used to assist in the development of clinical strategies to improve the long-term health of women.

## Acknowledgments

The chief acknowledgment is to the participants, the project staff, and the China National Centre for Disease Control and Prevention (CDC) and its regional offices for assisting with the fieldwork (Data Supplement). We thank Judith Mackay in Hong Kong; Yu Wang, Gonghuan Yang, Zhengfu Qiang, Lin Feng, Maigeng Zhou, Wenhua Zhao, and Yan Zhang in China CDC; Lingzhi Kong, Xiucheng Yu, and Kun Li in the Chinese Ministry of Health; and Sarah Clark, Martin Radley, Mike Hill, Hongchao Pan, and Jill Boreham in the Clinical Trial Service Unit, Oxford, for assisting with the design, planning, organization, and conduct of the study; and Fiona Bragg in University of Oxford for language checking and polishing.

## Sources of Funding

The China Kadoorie Biobank baseline survey and the first resurvey were supported by the Kadoorie Charitable Foundation in Hong Kong. The long-term follow-up is supported by the UK Wellcome Trust (088158/Z/09/Z, 104085/Z/14/Z), Chinese Ministry of Science and Technology (2011BAI09B01), Chinese National Natural Science Foundation (81390540, 81390544, 81390541), and National Key Research and Development Program of China (2016YFC0900500, 2016YFC0900501, 2016YFC0900504). The British Heart Foundation, UK Medical Research Council, and Cancer Research UK provide core funding to the Clinical Trial Service Unit and Epidemiological Studies Unit at Oxford University for the project.

## Disclosures

None.

## Supplementary Material
